# Supplementary material for: The Effects of Castration, Implant Protocol, and Supplementation of Bos indicus-Influenced Beef Cattle under Tropical Savanna Conditions on Growth Performance, Carcass Characteristics, and Meat Quality
Source: Animals (Basel). 2022 Feb 2;12(3):366. doi: 10.3390/ani12030366 (PMC8833375; doi:10.3390/ani12030366)
Supplement: Supplementary file 1 [file animals-12-00366-s001.zip › animals-1547255-supplementary.pdf]

**Table S1.** Experimental design indicating the distribution of observations (n) by breed type, implant protocol, and supplementation treatment for the sample of grazing bulls (Experiment I).

| Breed type | ZER-ZER[a] | | TBA/E2-ZER[b] | | Total |
|---|---|---|---|---|---|
| | SS[c] | MS [d] | SS[c] | MS [d] | |
| Brahman | 3 | 3 | 3 | 3 | 12 |
| F1-Angus | 3 | 4 | 3 | 6 | 16 |
| F1-Romosinuano | 3 | 5 | 3 | 5 | 16 |
| F1-Senepol | 4 | 5 | 2 | 5 | 16 |
| F1-Simmental | 4 | 5 | 3 | 5 | 17 |
| Brahman cross | 2 | 3 | 4 | 3 | 12 |
| ¾ *Bos taurus* | 3 | 3 | 2 | 2 | 10 |
| Total | 22 | 28 | 20 | 29 | 99 |

Source: Huerta-Leidenz *et al*. [35].

[a] ZER-ZER: corresponds to a dose of (72 mg) zeranol at day 0 followed by a second dose of 72 mg of zeranol at d-90;

[b] TBA/E2-ZER: corresponds to a first dose containing 140 mg trenbolone acetate + 20 mg estradiol 17β at d-0, followed by a second dose of (72 mg) of zeranol at d-90.

[C] Supplementation treatment: SS: strategic supplementation (SS); [d] MS: mineral supplementation as a control treatment.

**Table S2.** Composition of the forage supplements used in Experiment I.

| Composition | Supplement (SS) [1] | | |
|---|---|---|---|
| Ingredient, % DM basis | Strategic-Phase 1 | Strategic-Phase 2 | Mineral |
| Feather meal | 10.0 | 10.0 | - |
| Whole cottonseed | 0.00 | 49.9 | |
| Rice polish | 77.0 | 27.1 | |
| Cane molasses | 5.0 | 5.0 | |
| Mineral premix | 7.0 | 7.0 | |
| Ionophore[2] | 1.0 | 1.0 | |
| Nutrient | | | |
| ME, kcal/kg | 2.514 | 2.809 | |
| CP, % | 17.78 | 25.82 | |
| P, % | 1.07 | 0.79 | 12.0 |
| Ca, % | 0.12 | 0.17 | 24.0 |
| Mg, % | | | 1.5 |
| S, % | | | 1.0 |
| Mn, % | | | 0.50 |
| Zn, % | | | 0.75 |
| Fe, % | | | 0.50 |
| Cu, % | | | 0.20 |
| Co, % | | | 0.004 |
| I, % | | | 0.02 |
| Se, % | | | 0.004 |

Source: Huerta-Leidenz *et al*. [35]

[1]Strategic supplement-Phase 1 was supplied in the first 60 days of the trial; Strategic supplement-Phase 2 was administered manually from day 60 to day 182 (122 days in total) at a rate of 1 kg/animal/day. The mineral supplement was offered to the control (MS) group throughout the test with free access (80 g/animal/day). [2]Salocin® was used as the ionophore.
